# The course of psychiatric symptoms in older age bipolar disorder during the COVID-19 pandemic

**DOI:** 10.1186/s40345-022-00274-4

**Published:** 2022-12-06

**Authors:** Melis Orhan, Nicole Korten, Almar Kok, Dore Loef, Ralph Kupka, Sigfried Schouws, Patricia van Oppen, Annemiek Dols

**Affiliations:** 1 Geestelijke Gezondheidszorg (GGZ), InGeest Specialized Mental Health Care, Amsterdam, The Netherlands; 2 Department of Psychiatry, Amsterdam Public Health, Amsterdam University Medical Center, Vrije Universiteit, Amsterdam, The Netherlands; 3 Department of Epidemiology and Biostatistics, Amsterdam Public Health Research Institute, Amsterdam University Medical Center, Amsterdam, The Netherlands; 4 Altrecht GGZ, Utrecht, The Netherlands; 5 Amsterdam Neuroscience, Amsterdam, The Netherlands

**Keywords:** Bipolar disorder, COVID-19, Older adults, Depressive, Anxiety, Mania

## Abstract

**Background:**

The COVID-19 pandemic gives us the unique opportunity to study the course of psychiatric symptoms and resilience in older adults with bipolar disorder (OABD) whilst experiencing a collective long lasting stressor. The aim of this study was to investigate the course of depressive, manic and anxiety symptoms in OABD during the first six months of COVID-19 and how loneliness and mastery are associated with this course. Mastery is defined as the control one experiences over one’s life and environment. Resilience is defined as adaptation to challenging life conditions encompassing several aspects of personal resources.

**Methods:**

In April 2020 (n = 81), June 2020 (n = 66) and September 2020 (n = 51), participants were included from the Dutch Older Bipolars (DOBi) cohort study.

**Results:**

Depressive, manic and anxiety symptoms increased over all timepoints. Participants with a higher sense of mastery experienced a greater increase in depressive and anxiety symptoms. Loneliness did not interact with the course of these symptoms.

**Conclusions:**

OABD were resilient in the first months of COVID-19 outbreak, however depressive, manic and anxiety symptoms increased as the pandemic continued. Treatment strategies in coping with long lasting stressful events should include the focus on sense of mastery.

## Background

As of the beginning of 2020, the world is suffering from a global pandemic due to the outbreak of coronavirus SARS-CoV-2 (COVID-19). To reduce the spread of this virus, local governments set up measures, varying in strictness and timing. Restriction measures as proposed by the World Health Organization (Center for Disease Control and Prevention, World Health Organisation 2020) included staying at home as much as possible, prohibiting group activities, and closing many public facilities. Because older people were regarded as the group most at risk for suffering severely of COVID-19 outcomes, visiting them was discouraged and for many, professional health care was halted or limited. In the first year of the COVID-19 outbreak, no vaccine was available, thus these measures were strictly maintained in order to reduce the spread of the virus and to protect the most vulnerable groups in our society. Studies that investigated the effects of the COVID-19 outbreak show greater psychological distress in psychiatric patients when compared with healthy controls (Hao et al. 2020). However, when symptom levels in psychiatric patients were compared before and during the first months of the COVID-19 outbreak, changes were minimal or even negative in individuals with severe and chronic mental health disorders (Orhan et al. 2021a; Pan et al. 2021).

Several studies reported younger age as a risk factor for mental health problems amid COVID-19 in the general population (Kang et al. 2020; Huang and Zhao 2020). However, it was also found in the general population that mental health problems during the COVID-19 outbreak were more prevalent among older adults (Hossain et al. 2020). In community dwelling older people in the Netherlands, loneliness increased in the first 2 months after the implementation of the COVID-19 measures, while there was no difference in depressive and anxiety symptoms (Tilburg et al. 2021). Notably, personal losses, concerns about the pandemic, and a declined trust in societal institutions were associated with increasing mental health problems and emotional loneliness and not the frequency of social contacts (Tilburg et al. 2021). Loneliness can be defined as the evaluation of a discrepancy between the desired and the achieved network of relationships as a negative experience (Jong et al. 2006). It is therefore an important target for interventions, especially during the COVID-19 outbreak, since social isolation and loneliness increase older adults’ risk for anxiety, depression, cognitive dysfunction, heart disease and mortality (Brooke and Jackson 2020).

Since both an older age and pre-existing health problems are found to be risk factors for an increase in mental health symptoms during the COVID-19 pandemic, older adults with bipolar disorder (OABD) are particularly vulnerable for a decrease in wellbeing. However, a study conducted in younger adult patients with bipolar disorder (BD) also showed an initial increase of manic symptoms (Koenders et al. 2021). Nevertheless, in the study that we have conducted in April 2020, in the beginning of the COVID-19 pandemic, we found in our cohort of OABD, that they showed less depressive, manic and anxiety symptoms in the first month of the COVID-19 pandemic, when compared to three years earlier (Orhan et al. 2021a).This is in line with findings in community dwelling older people (Tilburg et al. 2021) and possibly a result of a ‘pulling together effect’ that can accompany an initial crisis (Reger et al. 2020). Hereby, individuals undergoing a shared experience might support one another, thus strengthening social connectedness and decreasing mental health symptoms. However, in patients with higher loneliness and lower mastery we found more depressive and anxiety symptoms. Therefore, we decided to focus on these associations in current follow-up study. Mastery is defined as the sense of control one experiences over one’s life and environment (Pearlin and Schooler 1978). Mastery is also associated with resilience (Skinner 1998), which is defined as the dynamic process of adaptation to challenging life conditions encompassing several aspects of personal resources and is considered to be protective against mental disorders (Kim-Cohen 2007). At this point, it remains unclear whether these patients also have a less favorable course of mental health symptoms during the COVID-19 pandemic. As this pandemic is a life-event that all patients are exposed to simultaneously, factors associated with recurrence of mental health symptoms during the COVID-19 outbreak may generalize to post-pandemic times and be identified as treatment targets.

The aim of this study was to investigate the course of depressive, manic and anxiety symptoms in OABD during the first six months of COVID-19 and how loneliness and mastery are associated with these symptoms. Our research questions are: (1) what is the course of depressive, manic and anxiety symptoms during the first 6 months of the COVID-19 pandemic, and (2) how are loneliness and mastery associated with the course of these symptoms during the COVID-19 pandemic? With the ongoing COVID-19 pandemic and associated measures, and thereby enduring exposure to a crisis environment and social isolation, we hypothesized that depressive, manic and anxiety symptoms will increase after the first months of the pandemic as the ‘pulling together effect’ fades off. We also hypothesize that participants who report more loneliness during the pandemic, will show a greater increase in depressive, manic and anxiety symptoms than participants who report less loneliness. In addition, we also hypothesize that participants that have a lower sense of mastery will show a greater increase in depressive, manic and anxiety symptoms.

## Methods

### Study sample

**Fig. 1:**
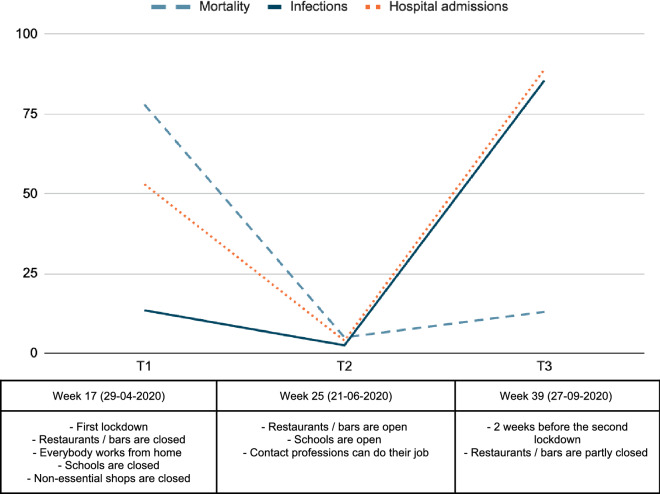
The number of hospital admissions and mortality during the COVID-19 pandemic in the Netherlands at T1, T2, and T3 (source: RIVM, 2021). T1 = April 2020; T2 = June 2020; T3 = September 2020; source: www.rivm.nl

Participants were recruited from the DOBi (Dutch Older Bipolars) cohort study. Participants had been included in the DOBi study in 2017 and 2018 (T0) (Dols et al. 2014). In brief, all patients aged 50 years and over in contact with services on January 1, 2017 were identified by a computerized search in the electronic record-keeping system of the Mental Health Organization (GGZ inGeest, Amsterdam, the Netherlands). Patients were screened for eligibility if they had any registered diagnosis of BD, which was confirmed in the Mini International Neuropsychiatric Interview (Sheehan et al. 1998). Medical records of all potential participants were screened by a psychiatrist for exclusion criteria (Dols et al. 2014). From the 130 Participants that were included in 2017 and 2018 (T0) 106 gave permission on their informed consent to contact them for follow-up studies (81.5%). Of these participants, 81 participated in April 2020, week 17 (T1), 66 participated in June 2020, week 25 (T2) and 51 participated in September 2020, week 39 (T3). Among all included participants, 50.6% participated in three evaluations, 37.3% participated in two evaluations and 12% participated in only one evaluation. Figure 1 shows the most important COVID-19 governmental measures, mortality rates, infections and hospital admissions on these dates in the Netherlands. DOBi was approved by the Medical Ethics Committee of the VU University Medical Center, Amsterdam, the Netherlands.

### Measurements

#### Demographics and mental health symptoms

Demographic data (e.g., age, gender, partner status) were obtained through interviews at T0, T1, T2, and T3, see Table 1. Mental health symptoms were measured at T0, T1, T2, and T3, respectively with the Young Mania Rating Scale (YMRS) (Young et al. 1978), with scores ranging from 0 to 60, and scores ≥ 12 indicating clinically relevant (hypo)mania, the Center for Epidemiologic Studies Depression Scale (CES-D) (Radloff 1977) with scores ranging from 0 to 60 and scores ≥ 16 indicating clinically relevant depression, and the Beck Anxiety Inventory (BAI) (Beck et al. 1988). The BAI is a 21-item self-report instrument for measuring the severity of anxiety; scores range from 0 to 63, whereby a score of 0–9 indicates normal or no anxiety, 10–18 mild to moderate anxiety, 19–29 moderate to severe anxiety and 30–63 severe anxiety.

**Table 1:** Psychiatric symptoms and social functioning at the different time points during the COVID-19 pandemic

	T0 (n = 81)	T1 (n = 81)	T2 (n = 66)	T3 (n = 51)
*Demographics*				
Age, M (SD)	–	66.1 (7.2)	66.4 (7.3)	66.9 (6.8)
Gender, female % (n)	55.6 (45)	55.6 (45)	50.8 (33)	54 (27)
*Living situation*				
Alone (%)	–	49.4	51.5	44.0
Children, yes %	–	50.6	50.0	48.0
Grandchildren, yes %	–	32.1	30.3	32.0
*Psychiatric symptoms*				
YMRS, median (IQR)	2 (4) 0–17	0 (3)	0 (2)	6 (3)
Above cut-off, %	1.2 (1)	1.2 (1)	0 (0)	0 (0)
CES-D, median (IQR)	12 (16.5) 0–51	8 (13.8)	11.5 (19)	13 (18)
Above cut-off, % (n)	53.5 (37)	26.2 (61)	43.4 (38)	59.2 (30)
BAI, median (IQR)	7 (15.3) 0–63	5.5 (7)	7 (12)	6 (14)
Above cut-off, % (n)	47.1 (32)	22.5 (18)	43.9 (29)	27.3 (14)
*Social functioning*				
Social participation M (SD)	23.4 (3.6)	16.6 (2.4)	19.2 (3.0)	20 (3.1)
Loneliness M (SD)	3 (6), 0–11	3 (4)	3.9 (3.2)	4.5 (3.2)
Mastery M (SD)	–	19.1 (5.2)	19.1 (5.1)	18 (8)
*COVID-19 related factors*				
COVID-19 infection, yes % (n)	–	1.2 (1)	1.5 (1)	3.9 (2)
Mental health impact, M (SD)	–	2.3 (0.8)	2.4 (0.8)	2.4 (0.7)
Fear for the virus, M (SD)	–	2.9 (0.6)	2.8 (0.7)	2.9 (0.6)
Positive coping, M (SD)	–	3.6 (0.6)	3.6 (0.7)	3.5 (0.6)

T1 = April 2020, T2 = June 2020, T3 = September 2020

YMRS, Young Mania Rating Scale; CES-D, Center for Epidemiologic Studies Depression Scale; BAI, Beck Anxiety Inventory; IQR, interquartile range; M, mean; SD, standard deviation

#### Loneliness

Feelings of loneliness were measured at T0, T1, T2, and T3. Loneliness was measured by the Loneliness Scale (De Jong Gierveld and Tilburg 1999). The scale has 11 items, with possible answers “yes”, “more or less” and “no”. The total loneliness score can be categorized into four levels with a score of 0–2 being not lonely, a score of 3–8 being moderately lonely, a score of 9–10 being severely lonely, and a score of 11 being very severely lonely.

#### Mastery

Mastery was measured at T1, T2, and T3. The Pearlin Mastery scale (Pearlin and Schooler 1978) measures the extent to which an individual regards their life chances as being under their personal control rather than fatalistically ruled. The scale has seven items, with answers on a 4-point Likert scale from “Strongly disagree (1)” to “Strongly agree (4)”. Scores range from 7 to 24, with higher scores indicating a higher sense of mastery.

### Statistical analysis

Means and standard deviations were computed to describe our sample at T0, T1, T2 and T3. In order to assess the course of symptoms of depression, anxiety and mania during the COVID-19 pandemic (T1, T2 and T3), we used linear mixed models with random intercept to compare changes in these measurements during COVID-19. Adding a random slope did not improve the model, so adding this was omitted. Missing data in depressive, manic and anxiety symptoms was imputed following multiple imputation, using age, loneliness and mastery at baseline and and psychiatric symptoms at all time points as predictors. Depressive, manic and anxiety were log-transformed in order to obtain a normal distribution. The models were estimated with random intercepts to account for the dependency in the data within individuals. We performed multiple independent linear-mixed effect models with depressive, manic and anxiety symptoms as dependent variables. The time points were dummy coded with T1 as comparator. Interaction effects were added to the model based on our earlier findings (Orhan et al. 2021a), with mastery and loneliness at T1 in separate models as main effect and interaction-effects, and age as confounding variable. Mastery and loneliness were divided by a median-split. Age was grand-mean centered in the analyses. Results with a p < 0.05 were regarded as statistically significant. Interaction-terms were considered statistically significant when p < 0.10.

## Results

### Course of mental health symptoms during the COVID-19 pandemic

#### Depressive symptoms

**Fig. 2:**
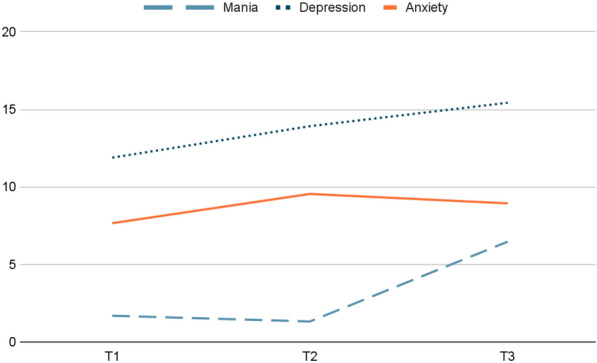
The course of psychiatric symptoms during the COVID-19 pandemic at T1, T2 and T3. T1 = April 2020, T2 = June 2020, T3 = September 2020. Depressive symptoms T1 = T2, T2 = T3, T1 < T3. Anxiety symptoms T1 < T2, T2 = T3, T1 = T3. Mania symptoms T1 = T2, T2 < T3, T1 < T3

Figure 2 shows the course of mental health symptoms during the first six months of the COVID-19 pandemic, whereas Table 2 presents the values of the associations between loneliness and mastery and the course of depressive, anxiety and mania symptoms. There was no significant difference in depressive symptoms between T1 and T2 (*b* = 0.17, *t* = 1.68, *p* = 0.10) and between T2 and T3 (*b* = 0.20, *t* = 1.86, *p* = 0.07). However, there was an overall significant increase in depressive symptoms between T1 and T3 (*b* = 0.37, *t* = 3.86, *p* < 0.01).

**Table 2:** Results of the linear mixed model analyses between different timepoints and psychiatric symptoms

	Depressive symptoms (log-transformed)			Anxiety symptoms (log-transformed)			Mania symptoms (log-transformed)		
Slope	Estimate	Std Error	p-value	Estimate	Std Error	p-value	Estimate	Std Error	p-value
T1-T2	0.17	0.10	0.10	0.18	0.09	0.04*	-0.08	0.10	0.45
T1-T2 * mastery	0.68	0.20	< 0.01**	0.59	0.17	< 0.01**	0.21	0.21	0.33
T1-T2 * loneliness	− 0.12	0.21	0.57	0.20	0.18	0.26	-0.14	0.21	0.51
T2-T3	0.20	0.11	0.07	− 0.11	0.12	0.33	10.17	0.08	< 0.01**
T2-T3 * mastery	− 0.34	0.21	0.11	-0.22	0.24	0.35	-0.19	0.16	0.23
T2-T3 * loneliness	− 0.17	0.21	0.41	-0.25	0.24	0.29	0.32	0.16	0.05*
T1-T3	0.37	0.10	< 0.01**	0.06	0.10	0.55	1.09	0.09	< 0.01**
T1-T3 * mastery	0.35	0.19	0.06	0.37	0.21	0.08	0.01	0.19	0.94
T1-T3 * loneliness	− 0.29	0.19	0.12	-0.05	0.21	0.80	0.18	0.18	0.32

T1 = April 2020, T2 = June 2020, T3 = September 2020

#### Anxiety symptoms

Anxiety symptoms increased significantly between T1 and T2 (*b* = 0.18, *t* = 2.05, *p* = 0.04). We did not observe a significant difference in anxiety symptoms between T2 and T3 (*b* = − 0.11, *t* = − 0.98, *p* = 0.33) or between T1 and T3 (*b* = 0.06, *t* = 0.60, *p* = 0.55).

#### Mania symptoms

Mania symptoms did not increase between T1 and T2 (*b* = − 0.08, *t* = − 0.75, *p* = 0.45). At T3, mania symptoms were significantly higher than at T2 (*b* = 1.17, *t* = 14.82, *p* < 0.01) and T1. (*b* = 1.09, *t* = 12.01, *p* < 0.01).

### Effect of mastery and loneliness on the course of mental health symptoms

**Fig. 3:**
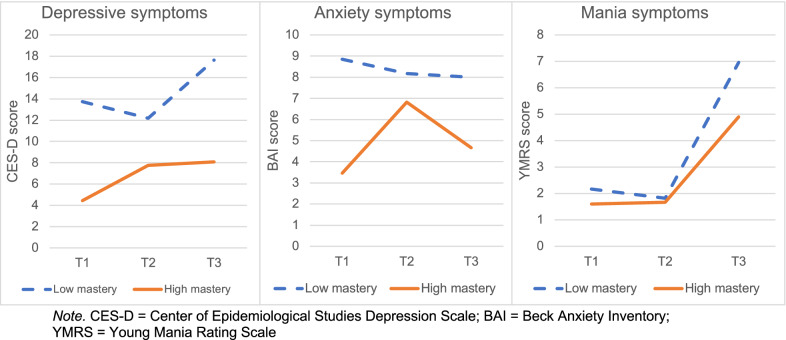
The course of depressive, anxiety and mania symptoms, separately presented for the low and high mastery group. CES-D, Center of Epidemiological Studies Depression Scale; BAI, beck anxiety inventory; YMRS, young mania rating scale

In Table 2 the interaction effects for mastery and loneliness on the course of the different mental health symptoms are shown. In Fig. 3 the significant interaction effects for mastery on the course of the different mental health symptoms are displayed.

#### The effect of mastery and loneliness on depressive symptoms

Between T1 and T2, the interaction effect for mastery and depressive symptoms was significant (*b* = 0.68, *p* < 0.01). Participants with a higher sense of mastery showed a greater increase in depressive symptoms than participants with a lower sense of mastery. Between T2 and T3, this effect faded (*b* = − 0.34, *p* = 0.11). In Fig. 3, mean differences between participants with high mastery and participants with low mastery are shown for each timepoint. Participants with higher mastery had lower levels of depression at each timepoint.

We did not find a significant interaction effect for loneliness between all the time points.

#### The effect of mastery and loneliness on anxiety symptoms

When looking at anxiety symptoms, we found a significant interaction effect for mastery and anxiety symptoms between T1 and T2 (*b* = 0.59, *p* < 0.01), with participants with a higher sense of mastery showing a greater increase in anxiety symptoms than participants with a lower sense of mastery between T1 and T2. We did not observe a significant interaction effect for mastery between T2 and T3 (*b* = − 0.22, *p* = 0.35). This indicates that there was no difference in increase in anxiety symptoms between patients with a higher and participants with a lower sense of mastery between T2 and T3. Participants with higher mastery had lower levels of anxiety at each timepoint.

For loneliness, we did not find a significant interaction effect between all the time points.

#### The effect of mastery and loneliness on mania symptoms

We did not observe a significant interaction effect for the sense of mastery between T1 and T2 (*b* = 0.23, *p* = 0.33) and between T2 and T3 (*b* = − 0.19, *p* = 0.23). This indicates that there was no significant difference in the course of mania symptoms in participants with different levels of mastery. Participants with higher mastery had lower levels of mania at each timepoint.

We did not find a significant interaction effect for loneliness and the course of mania symptoms between T1 and T2, (*b* = − 0.14, *p* = 0.51), T1 and T3 (*b* = 0.18, *p* = 0.32). However, we did find a significant interaction effect for loneliness between T2 and T3 (*b* = 0.32, *p* = 0.05). This interaction effect indicates that participants who experience more loneliness also show a greater increase in mania symptoms between T2 and T3.

## Discussion

This is the first study investigating the course of depressive, manic and anxiety symptoms in OABD during the COVID-19 pandemic. The results of our study show that these symptoms were of relatively low intensity in the first month of the pandemic, but they increased in six months as the pandemic continued. Mastery seemed to be a significant effect modifier of changes for depressive and anxiety symptoms, whereas loneliness did not interact with the course of psychiatric symptoms.

In line with our hypothesis; depressive and mania symptoms increased during the whole period and anxiety symptoms only increased in the first three months of the pandemic, and stayed relatively stable thereafter. In this period, the Netherlands experienced several months of lockdown, with closing of almost all facilities and without a solution in prospect. This increase in symptoms might be an effect of diminishing of the earlier mentioned ‘pulling together’ effect (Reger et al. 2020). Our study is in line with a study conducted in older adults with pre-existing depressive symptoms during COVID-19 (Hamm et al. 2020). In this study, it was observed that during the first two months of the COVID-19 pandemic participants were doing relatively well, but most of the participants forecasted that their mental health would deteriorate as the COVID-19 measures continued. This finding is supported by an often experienced emotionally positive “honeymoon phase” of the disaster response (Math et al. 2006). This concept has been used to describe resilient psychological responses directly following acute disasters, including community bonding and optimism that everything will return to normal quickly. After the “honeymoon phase”, the “disillusionment phase” enters. This phase might be represented by the increase in depressive, manic and anxiety symptoms in our group, since this phase includes optimism turning into discouragement and stress concerning the situation increases.

Besides the course of mental health symptoms, we also studied the effect of the sense of mastery on this course. Mastery is seen as a psychological coping resource and has been recognized as an indicator of resilience (Skinner 1998). In our sample, we found that in the first three months of the pandemic, participants with a higher sense of mastery showed a greater increase in depressive and anxiety symptoms than participants with a lower sense of mastery. However, participants with a higher sense of mastery still showed less psychiatric symptoms, which is in line with an earlier study in younger adult patients that showed that a higher sense of mastery was associated with less depressive symptoms during COVID-19 (Orhan et al. 2021a).

Additionally, a study on the effects of accumulation of negative life events on depressive symptoms in old age, it was found (Kok et al. 2021) that the detrimental effect of recent life events on mental health was weaker for persons who had previously been exposed to more negative events. However, this ‘steeling’ effect was stronger in persons with *lower* mastery. It is possible that COVID-19 related stressors, including quarantine, fear and loss of loved ones, lead to learning those who felt strongly in control of their lives, that circumstances can actually arise that one cannot control. This might have learned that their more active coping style, fell short in this specific situation. A more passive coping, and thereby having a lower sense of mastery, could be more adequate in this situation. The more passive use of acceptance in combination with novelty seeking as main coping strategies can be useful in chronic circumstances that one has no control over (Schouws et al. 2015). A high sense of mastery can thus be regarded as non-beneficial when circumstances arise that one cannot control, where it might be more beneficial to accept this and to seek pleasure in other aspects of life. However, in the long run, mastery might contribute to better resilience.

We also found that the initial negative effect of loneliness on mental health symptoms, did not persist after the first three months of the pandemic. In a study conducted in community-dwelling older adults it was found that they experienced an increase in loneliness in the first two months of the pandemic, but mental health remained roughly stable (Tilburg et al. 2021). In our earlier study (Orhan et al. 2021a), we have found that loneliness was cross-sectionally associated with depressive symptoms. However, by conducting analyses on interaction effect, we studied whether the increase in symptoms was greater in participants that had high loneliness at T1, when compared to participants that had low loneliness at t1. We did not find any significant interaction effects. However, post-hoc analyses. Revealed that participants that already showed the highest loneliness scores at baseline (highest quartile), also had higher depression scores than the other participants (median = 15 vs. median = 8). Therefore, a greater increase was not to be expected. Thus, loneliness is associated with mental health symptoms (Heinrich and Gullone 2006) but during the pandemic it was not a risk factor for a (further) increase in depressive, manic and anxiety symptoms. This is in line with findings in the general Dutch population, that suggest that the pandemic did not negatively affect the prevalence of anxiety and depression during the first four months, but that loneliness did increase (Kok et al. 2022). It was also found in patients with pre-existing psychiatric symptoms, that there was not a strong increase in symptoms during the COVID-19 pandemic in those with a higher burden of disorders. In fact, changes in scores from before to during the pandemic, indicated increased symptom levels in people without psychiatric disorders whereas this was not found in participants with more chronic psychiatric disorders (Van der Velden et al. 2022).

Our study has several strong points. First, we were able to collect data on different timepoints during this global pandemic. From a scientific perspective, this pandemic offers a unique possibility to study the course of mental health symptoms and risk factors for adverse outcome during a collective negative life event. Despite these strong points, there are also some limitations that need to be acknowledged. We have included a relatively small group of participants, therefore statements about generalizability should be made with caution. Next, our data were collected in the first six months of the pandemic, thus our findings might not reflect the long-term effects of the COVID-19 pandemic.

The results of our study warrant clinical implications. Clinicians need to be aware of a possible increase of mental health symptoms during a global life event, such as a pandemic and the possible role of inadequate coping strategies as these situations continue. We found that mastery might be beneficial on the short-term, but when uncontrollable events happen, mastery might not be the most beneficial coping style. In addition, it deserves to be stressed that OABD are not experiencing disproportionately increased mental health symptoms, regarding that most participants still do not experience symptoms above the cut-off score. This was also the case for participants that were included at T1, but were lost to follow-up. However, we observed an increase in depressive, manic and anxiety symptoms and therefore this group needs to be carefully monitored as the pandemic continues. In order to prevent further increase of symptoms, clinicians can focus on teaching more adequate coping strategies, e.g., by learning cognitive behavioral therapy (CBT) techniques. CBT aims to improve patients’ ability to cope with their illness and possibly their sense of mastery (Henken et al. 2020). CBT is a relatively short-term, focused treatment for many types of psychiatric disorders that helps individuals to identify dysfunctional thoughts, attitudes, and behaviors and learn healthier skills and habits (Beck 2011). Our study shows a disadvantageous effect of higher mastery for the course of depressive and anxiety symptoms during the COVID-19 pandemic. However, participants with higher sense of mastery still reported less psychiatric symptoms during the COVID-19 pandemic. A suggestion for future research might therefore be looking more closely into the concept of mastery and its effect on mental health symptoms.

## Conclusions

In conclusion, the COVID-19 pandemic gives us the unique opportunity to study the course of mental health symptoms in OABD whilst experiencing a collective negative life event. OABD were resilient in the first months of COVID-19 outbreak, but mental health symptoms increased as the pandemic continued. Mastery seemed to be a factor that interacted with the course of depressive and anxiety symptoms. This stresses the need to focus on prevention strategies for recurrence in this vulnerable group and to include attention for the sense of mastery as an important part of treatment strategies.

## Data Availability

The data (Orhan et al., 2021b) that support the findings of this study are available from the corresponding author upon reasonable request.

## Abbreviations

COVID-19: Coronavirus SARS-CoV-2
OABD: Older adults with bipolar disorder
BD: Bipolar disorder
T1: Timepoint 1
T2: Timepoint 2
T3: Timepoint 3
DOBi: Dutch Older Bipolars cohort
CES-D: Center of Epidemiological Studies Depression Scale
YMRS: Young Mania Rating Scale
BAI: Beck Anxiety Inventory
CBT: Cognitive behavioral therapy
IQR: Interquartile range
M: Mean
SD: Standard deviation

## References

[CR1] Beck JS (2011). Cognitive-behavioral therapy. Clinical textbook of addictive disorders.

[CR2] Beck AT, Epstein N, Brown G, Steer RA (1988). An inventory for measuring clinical anxiety: psychometric properties. J Consult Clin Psychol.

[CR3] Brooke J, Jackson D (2020). Older people and COVID-19 isolation, risk and ageism. J Clin Nurs.

[CR4] Center for Disease Control and Prevention, World Health Organisation. Mental Health and Coping during COVID‐19. Atlanta, GA: CDC; 2020.

[CR5] De Jong Gierveld J, Van Tilburg T (1999). Manual of the loneliness scale.

[CR6] de Jong Gierveld J, Van Tilburg T, Dykstra PA (2006). Loneliness and social isolation. Cambridge handbook of personal relationships.

[CR7] Dols A, Rhebergen D, Beekman A, Kupka R, Sajatovic M, Stek ML (2014). Psychiatric and medical comorbidities: results from a bipolar elderly cohort study. Am J Geriatr Psychiatr.

[CR9] Hamm ME, Brown PJ, Karp JF, Lenard E, Cameron F, Dawdani A (2020). Experiences of American older adults with pre-existing depression during the beginnings of the COVID-19 pandemic: a multicity, mixed-methods study. Am J Geriatr Psychiatry.

[CR10] Hao F, Tan W, Jiang L, Zhang L, Zhao X, Zou Y (2020). Do psychiatric patients experience more psychiatric symptoms during COVID-19 pandemic and lockdown? A case-control study with service and research implications for immunopsychiatry. Brain Behav Immun.

[CR11] Heinrich LM, Gullone E (2006). The clinical significance of loneliness: a literature review. Clin Psychol Rev.

[CR12] Henken HT, Kupka RW, Draisma S, Lobbestael J, Van Den Berg K, Demacker SMA, Regeer EJ (2020). A cognitive behavioural group therapy for bipolar disorder using daily mood monitoring. Behav Cogn Psychother.

[CR13] Hossain MM, Tasnim S, Sultana A, Faizah F, Mazumder H, Zou L (2020). Epidemiology of mental health problems in COVID-19: a review. F1000.

[CR14] Huang Y, Zhao N (2020). Chinese mental health burden during the COVID-19 pandemic. Asian J Psychiatr.

[CR15] Kang C, Tong J, Meng F, Feng Q, Ma H, Shi C (2020). The role of mental health services during the COVID-19 outbreak in China. Asian J Psychiatr.

[CR16] Kim-Cohen J (2007). Resilience and developmental psychopathology. Child Adolesc Psychiatr Clin N Am.

[CR17] Koenders M, Mesbah R, Spijker A, Boere E, de Leeuw M, van Hemert B, Giltay E (2021). Effects of the COVID-19 pandemic in a preexisting longitudinal study of patients with recently diagnosed bipolar disorder: Indications for increases in manic symptoms. Brain Behav.

[CR18] Kok AA, Twisk JW, Blom F, Beekman AT, Huisman M (2021). Steeling or sensitizing? A longitudinal examination of how ongoing accumulation of negative life events affects depressive symptoms in older adults. J Gerontol B Psychol Sci Soc Sci.

[CR19] Kok AA, Pan KY, Rius-Ottenheim N, Jörg F, Eikelenboom M, Horsfall M (2022). Mental health and perceived impact during the first Covid-19 pandemic year: a longitudinal study in Dutch case-control cohorts of persons with and without depressive, anxiety, and obsessive-compulsive disorders. J Affect Disord.

[CR20] Math SB, Girimaji SC, Benegal V, UdayKumar GS, Hamza A, Nagaraja D (2006). Tsunami: psychosocial aspects of Andaman and Nicobar islands. Assessments and intervention in the early phase. Int Rev Psychiatry.

[CR21] Orhan M, Korten N, Paans N, de Walle B, Kupka R, van Oppen P (2021). Psychiatric symptoms during the COVID-19 outbreak in older adults with bipolar disorder. Int J Geriatr Psychiatry.

[CR22] Orhan M, Korten N, Dols A. DOBI_COVID_volledig; GGZ inGeest/Amsterdam UMC. 2021b.

[CR23] Pan KY, Kok AA, Eikelenboom M, Horsfall M, Jörg F, Luteijn RA (2021). The mental health impact of the COVID-19 pandemic on people with and without depressive, anxiety, or obsessive-compulsive disorders: a longitudinal study of three Dutch case-control cohorts. Lancet Psychiatry.

[CR24] Pearlin LI, Schooler C (1978). The structure of coping. J Health Soc Behav.

[CR25] Radloff CS (1977). The CES-D scale: a self-report depression scale for research in the general population. Appl Psychol Meas.

[CR26] Reger MA, Stanley IH, Joiner TE (2020). Suicide mortality and coronavirus disease 2019—a perfect storm?. JAMA Psychiat.

[CR27] Schouws SN, Paans NP, Comijs HC, Dols A, Stek ML (2015). Coping and personality in older patients with bipolar disorder. J Affect Disord.

[CR28] Sheehan DV, Lecrubier Y, Sheehan KH (1998). The mini international neuropsychiatric interview. (M.I.N.I): the development and validation of a structured diagnostic psychiatric interview for DSM-IV and ICD-10. J Clin Psychiatr..

[CR29] Skinner EA (1998). A guide to constructs of control. J Pers Soc Psychol.

[CR30] Van der Velden PG, Hyland P, Contino C, von Gaudecker HM, Muffels R, Das M (2021). Anxiety and depression symptoms, the recovery from symptoms, and loneliness before and after the COVID-19 outbreak among the general population: findings from a Dutch population-based longitudinal study. PloS ONE..

[CR31] Van Tilburg TG, Steinmetz S, Stolte E, van der Roest H, de Vries DH (2021). Loneliness and mental health during the COVID-19 pandemic: a study among Dutch older adults. J Gerontol B Psychol Sci Soc Sci.

[CR32] Young RC, Biggs JT, Ziegler VE, Meyer D (1978). A rating scale for mania: reliability, validity and sensitivity. Br J Psychiatry.

